# Mastery Without Mystery: Why there is no Promethean Sin in Enhancement

**DOI:** 10.1111/j.1468-5930.2011.00543.x

**Published:** 2011-11

**Authors:** Guy Kahane

**Affiliations:** Oxford Uehiro Centre for Practical Ethics, Faculty of PhilosophyLittlegate House, St. Ebbe's Street, Oxford OX1 1PT, UK. guy.kahane@philosophy.ox.ac.uk

## Abstract

Several authors have suggested that we cannot fully grapple with the ethics of human enhancement unless we address neglected questions about our place in the world, questions that verge on theology but can be pursued independently of religion. A prominent example is Michael Sandel, who argues that the deepest objection to enhancement is that it expresses a Promethean drive to mastery which deprives us of openness to the unbidden and leaves us with nothing to affirm outside our own wills. Sandel's argument against enhancement has been criticized, but his claims about mastery and the unbidden, and their relation to religion, have not yet received sufficient attention. I argue that Sandel misunderstands the notions of mastery and the unbidden and their significance. Once these notions are properly understood, they have surprising implications. It turns out that the value of openness to the unbidden is not just independent of theism, as Sandel claims, but is in fact not even fully compatible with it. But in any case that value cannot support Sandel's objection to enhancement. This is because it is not enhancement but certain forms of opposition to enhancement that are most likely to express a pernicious drive to mastery.

## 1. Religious Sentiments Without Religion?

When we confront scientific advances that might allow us to radically reshape human nature, familiar ethical concepts and categories can seem woefully inadequate. As Michael Sandel writes,

In order to grapple with the ethics of enhancement, we need to confront questions largely lost from view — questions about the moral status of nature, and about the proper stance of human beings toward the given world. Since these questions verge on theology, modern philosophers and political theorists tend to shrink from them.^1^

These sentiments echo earlier remarks by Ronald Dworkin and Jürgen Habermas.^2^

I agree, and have argued elsewhere,^3^ that recent philosophy has neglected important questions about value — questions that are not about wellbeing, autonomy or justice but about what attitude we should have to the world and our place in it.^4^ These are questions we must ask even if we are not religious believers.

It is natural to call such questions *theological*, *religious* or *spiritual* in a sense that doesn't imply acceptance of any religion. You don't need to be a believer to exhibit what Wittgenstein and Thomas Nagel call a *religious point-of-view* or *temperament*.^5^ But such language is misleading. Such questions are not literally theological, since they don't presuppose the truth of theism. And they cannot be literally religious or spiritual because they require no religion, and can be asked even by the most uncompromising materialist. This is why I will instead speak of *existential* questions, attitudes and values.^6^

Such existential or ‘religious’ values are often invoked to reassure non-religious conservatives that they can join religious believers in opposing, say, human enhancement, abortion, or homosexuality. The idea is that it can be legitimate to give moral weight to certain religious attitudes, values or practices even if one doubts the metaphysical substance of religious belief.^7^

It is natural to be suspicious about such suggestions — after all, the defenders of ‘intelligent design’ also insist, disingenuously, that their claims have nothing to do with religion. Nevertheless, it *is* possible that certain important values and attitudes associated with some religions do not in fact require the existence of God. We can call these *theism-neutral* existential values. Such values would need to be independent not only of belief in God, but of any kind of revelation, mystical experience or sacred text.

Existential attitudes are attitudes we ought to have towards the world. Such attitudes are invoked when, in a key passage, Sandel writes that:

… the deepest moral objection to enhancement lies less in the perfection it seeks than in the human disposition it expresses and promotes … The problem is in the hubris of the designing parents, in their drive to master the mystery of birth … it would disfigure the relation between parent and child, and deprive the parent of the humility and enlarged human sympathies that an openness to the unbidden can cultivate.^8^

In another passage, Sandel explains that:

… the deeper danger is that [enhancement] represents a kind of hyperagency — a Promethean aspiration to remake nature, including human nature, to serve our purpose and satisfy our desires … And what the drive to mastery misses and may even destroy is an appreciation of the gifted character of human powers and achievements.^9^

Sandel describes these worries as expressing a ‘religious sentiment’, but he insists that it's a sentiment that resonates ‘beyond religion’— in other words, Sandel means the values he invokes to be *theism-neutral*,^10^ although his critics nevertheless often suspect that they are just religion in disguise.

Unfortunately, these and similar passages in Sandel are so opaque that it is hard to assess the worry, or be clear about its relation to religion. One way to respond to Sandel's worries about enhancement is to ignore these larger sentiments and consider instead whether his specific normative claims about enhancement are consistent or plausible.^11^ But although these passages are opaque, they also raise interesting philosophical issues, and the suggestion that there are important and neglected values and attitudes that originate in religion but resonate beyond it deserves close attention.

In this essay I will begin to explore this intriguing suggestion. I will do so by trying to make sense of a central strand in Sandel's objection to enhancement: the complaint that it expresses a disfiguring drive to mastery, and would undermine our appreciation of the unbidden.^12^ Now, it is possible that genetic engineering or cloned sheep first draw our attention to the flaw in mastery, or the value of the unbidden. But if these are genuine values, they should have a hold in *all* corners of life, and we should therefore first try to understand them independently of speculations about future technologies. This is how I will proceed: with general reflections on mastery and the unbidden. I will turn to reproduction, and enhancement, only at the end.

## 2. Mastery and the Unbidden

We can start with the distinction between chance and choice: between the bidden and unbidden, or, put differently, between what we have mastered, and what we have not, or cannot.

This distinction is often misunderstood. Chance in this sense is simply what is outside our control.^13^ This *needn't* imply randomness, or unpredictability.^14^ It is unsurprising, but still unbidden, that the sun rises every morning.^15^

Talk about the unbidden is ambiguous in another sense. If a distinguished speaker cancels an appearance at an event at the last moment, this unpleasant surprise is utterly unbidden for the organizers, but it is nevertheless also a matter of choice. It is a rather different thing for something to be unbidden, not *relative* to someone, but *absolutely*— for it to occur independently of *anyone's* agency.

Now consider this: if God exists, then *nothing* is absolutely unbidden.^16^*Nothing* happens that doesn't have its source in some agency. There is *always* some agent that is ultimately responsible for everything that happens.^17^ The absolutely, unqualifiedly unbidden exists *only* in a naturalist, Godless universe. Indeed, in such a universe nearly everything that happens is absolutely unbidden. It simply occurs, without meaning or purpose.^18^

When something occurs, whether bidden or unbidden, it can be good, bad or indifferent. If its occurrence is under our control then (so long as we aim at the good) the outcome is more likely to be good than if left to chance. This is why, when we value something, we should try, when possible and permissible, to bring it about. Given this truism, it seems odd to think that, when something matters, we should ever leave it to chance rather than choice.

Some theists would reject this conclusion. If God exists, then nothing is unqualifiedly unbidden, due *purely* to chance. Perhaps everything that happens plays some role in a divine plan — even if this plan is inscrutable to us mortals. And this might mean that we sometime have reason to just let the dice fall where they may. This religious belief can have extreme implications. For example, the Moravian Church, an evangelical Protestant movement, held at one point that all important decisions should be decided by chance — they even used lots to decide whether some couple should marry.^19^

Whether or not such practices are theologically defensible,^20^ they do not involve genuine ‘openness to the unbidden’. For these believers assumed precisely that lots are *not* decided by pure chance, but express God's *good will*.^21^ Many religious traditions tell us, in similar ways, to resign ourselves to fate, however grim. But again, to say, ‘Thy will be done’ is not to be open to the absolutely unbidden, but to think of hardship and ill as part of some larger beneficent whole, or as compensated by later, transcendent reward.^22^

Theist religions may encourage humility, and submission to God's will. But they do not encourage openness to what is *absolutely* unbidden.

The Judeo-Christian tradition certainly doesn't exalt pure chance. Heaven isn't portrayed as a realm of constant risk and surprise. And when the ancients worshipped Fortuna, the goddess of chance, this wasn't because they celebrated the unbidden, butbecause they feared it, and pathetically hoped to control it. It is instructive here to remember Augustine's question:

How, therefore, is she good, who without discernment comes to both the good and to the bad? … It profits one nothing to worship her if she is truly *fortune* … let the bad worship her … this supposed deity.^23^

Our understanding of the unbidden, and our attitude to mastery, thus *directly* depend on whether we believe that God exists. For if He *doesn't* exist, then there is no master plan, and what we leave to chance we *really* leave to purposeless chance. So why should we just let things happen, when it's in our power to make them better?^24^ To leave things to chance would suggest that they don't really *matter* to us. So, at least for naturalists, there is a clear rational presumption in favour of mastery.^25^

There is, then, a profound difference between accepting whatever happens because it expresses God's good will, and accepting it *simply because it happens*— between accepting a providential plan that is unbidden only *relative* to us, and accepting what is *absolutely* unbidden.

Now if God exists, it might be a virtue to exhibit humility and submission to His will. But this value makes no sense if God doesn't exist. It cannot ground a theism-neutral objection to mastery. So what else could be wrong with mastery? In the closing words of his book, Sandel writes that the drive to mastery threatens ‘… to leave us with nothing to affirm or behold outside our own will’.^26^ This worry confuses mastery with something else. What Sandel has in mind here is a kind of romantic wilfulness or self-assertion, the rejection of any limit to the will, or of anything external to self. Perhaps even the wish to go beyond good and evil — perhaps even to create oneself. In *Paradise Lost*, Milton famously depicted Satan in this light, as knowingly rejecting goodness. In modern versions, such wilfulness is often associated with a kind of nihilism. (Some religious believers like to portray atheism as driven by (or at least implying) such nihilistic wilfulness. This is a risible caricature which, unfortunately, and tellingly, Sandel's criticism of enhancement echoes.)

The desire for absolute mastery or god-like omnipotence is a childish fantasy. No one will ever find himself with literally ‘nothing to affirm or behold outside their own will.’ The idea of literally creating oneself is simply logically incoherent. Nor can we change the laws of nature or the past, or shape logic, value or morality to match our whims. And human powers are embarrassingly feeble. We have a measure of mastery over a narrow domain on the surface of little planet Earth. But we cannot even predict, let alone control, tomorrow's weather.

In any case, mastery in the sense I described earlier hardly expresses a failure to acknowledge anything outside our will. On the contrary: we can *only* master what is external to our will,^27^ and mastery is typically an achievement that makes us intimately aware of a resistant world. Nor does it express brute self-assertion: to aim to produce good is precisely to be subservient to external standards of value. Indeed, it's not just that the unbidden isn't the same as the unpredictable, as Sandel assumes. It's that the *necessary* is the most *profound* example of the unbidden. We cannot make two plus two equal five — not even God can!^28^ And what morality dictates, and what is good or bad, are similarly immutably independent of our passing whims.^29^

We mustn't confuse mastery with wilfulness. But Sandel's fear that we might find ourselves with ‘nothing to affirm … outside our own will’ does gesture at something genuine — the important idea that what is truly real is what is external to, and resistant, to our will. This idea has a long history. Fichte argued that a self-conscious finite being necessarily requires something external (a ‘not-I’) that is opposed to it.^30^ Freud famously distinguished between the pleasure principle and reality principle.^31^ Versions of this idea surface in Cardinal Newman and Simone Weil, and more recently, in Robert Nozick, Harry Frankfurt and Bernard Williams.^32^

In its simplest form, it concerns the struggle between belief and desire — the difficulty of attaining a view of things that isn't distorted by wishful thinking and self-deception. But there is a more subtle way in which we might lose touch with reality. Not when our beliefs reflect our wishes, but when the world itself instantly reflects them. Milan Kundera writes that:

[t]he heavier the burden … the more real … [our lives] become. Conversely, the absolute absence of a burden causes man to be lighter than air, to soar into the heights … and become only half real, his movements free as they are insignificant.^33^

Think of a spoiled child, whose every wish is immediately granted. The world around him is still deeply resistant to the will, as he will soon find out. But he has lost sight of that fact, and his tantrums precisely express his rejection of any external limit.^34^ This is another way in which the distinction between fact and fantasy can be blurred, and the world can lose its reality. (Though if to have things reflect one's will is to lose grip of the world, then doesn't it follow that for God the whole universe is nothing but a dream?)

For things to be resistant to our will, they don't need to be absolutely unbidden. It is enough that they express the will of others, whether human or divine. Indeed we experience the unbidden most acutely when others violently impose their will on us. Jean Amery wrote of Auschwitz that ‘[n]owhere else was reality so real’.^35^

Conversely and far more benignly, we acknowledge the existence of something external to our will whenever we respect the wills of *others*. This is a foundation of morality: in familiar ways, we often have reason to contract our mastery and allow others to make their own choices, for good or bad, rather than paternalistically impose our will on their lives. This is of course simply the value accorded to autonomy in the Kantian tradition. Indeed one might say that in being open to the unbidden of others' wills, we are also respecting their capacity for *mastery*. (Again: mastery and appreciation of the unbidden are not opposed; they are complementary.)

The existence of other persons, then, already provides a sense of the limits of our mastery. Many religious believers would argue that this isn't enough. To fully appreciate that there is something external to us, we need something that is external to *all* of us, something profoundly different and other. Such appreciation, they will add, can only come when we acknowledge and worship a supernatural, divine being.

I want to suggest a contrary view. We can fully appreciate something truly external to us only when we confront the vastness of an utterly impersonal and purposeless universe — that is to say, when we confront what I have called the *absolutely unbidden*.

Nothing is absolutely unbidden in this way if God exists. Nothing is truly alien. Theists hold that humans were created in God's image. Many atheists think that God is a *projection* of the human. Atheists, I suggest, could go further, and understand religious belief as a way of avoiding genuine acceptance of the unbidden.^36^ After all, God is seen as a source of hope for the fulfilment of our deepest wishes. Religion promises us that, despite appearances, there is a deep harmony between our needs and the world, and everything is meaningful. Or take belief in the afterlife: Death is the ultimate unbidden, a final limit on the will — a limit that many find incredibly hard to accept. (In fact, there is intriguing psychological evidence showing that when people feel less in control, they are *more* likely to affirm belief in God — and, indeed, in magic.^37^)

To face a universe that is truly unbidden is not comforting in these ways. It is an experience of anxiety and alienation, not of unity and harmony. Genuine acceptance of the absolutely unbidden is difficult even for non-believers; we constantly anthropomorphize the inhuman universe the surrounds us. It's a real achievement to fully abstract from the human perspective and genuinely confront the thing in itself — what Wallace Stevens called ‘mere being’.^38^ For example, when Richard Dawkins describes the universe as ‘nothing but blind, pitiless indifference’,^39^ he is descending to metaphor. Even atheists, then, have to be on guard against the temptation to idolatry.^40^ To think of the absolutely unbidden as literally a *gift* to which we owe gratitude is, I believe, to succumb to this temptation.^41^

Sandel portrays himself as appealing to existential values that, although they derive from religious sentiment, resonate beyond it. But the value of the unbidden isn't really theism-neutral. Not, as some of Sandel's critics think, because it *presupposes* the truth of some religion. Quite the contrary. We saw that existential attitudes and values can be dependent on theism, or neutral with respect to it. But once we stop thinking in terms of ‘theological’ values, we can see that there is a third possibility. There might also be existential values that are distinctly *atheist*: attitudes that are appropriate, and values that can be realized without qualification (or even at all) only if God *doesn't* exist. If the unbidden is valuable, it is an example of such a value: it can only be fully realized in a Godless world.^42^

Since the idea of complete mastery is incoherent, there's no worry that the unbidden would someday disappear from our lives. But we can perhaps worry that, like the spoiled child, we might fail to properly appreciate it. And perhaps modern technology can stand in the way of such appreciation.^43^ We should guard against this danger. But to appreciate the unbidden needn't mean just letting things happen. To maturely recognize the limits to our agency, to learn to accept what we can't change, isn't at all the same as imposing arbitrary limits on our agency, or accepting what we *can* change.

In fact, the idea that we should act to preserve the unbidden, perhaps by forbidding the development of certain technologies, comes close to being self-defeating, since such acts are *themselves* instances of mastery. Kundera asks: ‘What … shall we choose? Weight or lightness?’ But to choose to master chance is still to choose, even if at a second-order level.^44^ Somewhat paradoxically, the value of the unbidden is one that we can fully respect only with complete passivity.

As one probes deeper, one begins to suspect that Sandel's worries aren't really about the unbidden, but about accepting one's place in some cosmic order. It is only against this *religious* picture that it could make sense to show humility towards the world, or that Promethean ‘hubris’ could be seen as a vice.

If there were such a cosmic hierarchy, then it would perhaps be wrong for us to trespass on God's grounds; perhaps we should stick to our human station and its duties. We should exhibit, not openness to the unbidden, but openness — or rather submission — to God's bidding.

But there is no cosmic hierarchy. If God doesn't exist, then one deep fact about our place in the universe is that we don't, in *this sense*, have a place in the universe.^45^ It makes no sense to worry that we are being uppity to the angels or God above (nor that we might offend Granny Nature). The universe isn't going to punish us for aiming too high. To think otherwise is just a servile superstition.

It might be objected that what Sandel has in mind is not some divinely ordained order, but a purely *natural* order — the view that how we ought to live, and what we are permitted to do, is somehow dictated by what is natural for human beings. It is true that such an understanding of nature doesn't *logically* require acceptance of theism. But it is also true that *without* theism, it is simply implausible.^46^ It becomes even less plausible once we attempt to square it with modern biology. After all, we are the contingent, unbidden products of natural selection, a process that is driven by reproductive fitness, not by the good.^47^ Attempts to revive such a pre-modern understanding of nature have been repeatedly subjected to devastating criticism,^48^ criticism that Sandel never acknowledges, let alone addresses. It would therefore be most disappointing to discover that his opposition to enhancement ultimately rests on no more than this discredited view. But there is another reason why I have simply ignored this view. For if there *were* a natural order that we ought to follow (whether or not it's divinely ordained) then appeals to the unbidden, and denunciations of mastery, would be simply *redundant*.^49^

## 3. Reproduction and the Genetic Lottery

In natural reproduction, genetic material from the parents is randomly combined to create the unique genetic endowment of the resulting child. In the future, to an extent we cannot yet predict, reproductive technologies might allow us to select at least some aspects of the characteristics of future children. To do so, Sandel argues, would be deeply wrong, because such mastery would undermine our openness to the unbidden. Reproduction should remain a mystery, unpredictable and outside human control.

An immediate problem with this argument is that natural reproduction is actually not so unpredictable. Parents expect and value expected similarities between themselves and their children. And, of course, they can control who they reproduce with, and when. Needless to say, birth control is a form of *control*.

We could change all of that. For example, instead of wilfully selecting whom we marry this could be decided by lottery.^50^ Instead of letting couples decide if and when to reproduce, contraception could be made mandatory — but with random flaws so that conception is always possible, but never predictable. (That is guaranteed to open people to the unbidden!) We could go even further, and replace the highly limited genetic lottery with a proper lottery, so that it will be impossible to predict what our children will be like: black or white, tall or short, handsome or ugly.

We could, in these ways, increase the role of chance in our lives. But I trust that no one thinks that the relation between parents and children is disfigured because we don't follow these proposals.^51^

Natural reproduction, then, isn't completely unbidden. But it's also important not to exaggerate the mastery we would come to possess if we did engage in genetic enhancement.^52^ Sex selection is already possible, but it's by no means obvious that it will ever be feasible to select for intelligence. Genetics is incredibly complex, and there is a gulf between genotype and phenotype. Enhancement will inevitably be a matter of calculating probabilities, which will get extremely complex when genes interact with an unpredictable environment. Only someone in the grip of a crude genetic determinism could worry that genetic selection would simply erase the unbidden from reproduction.^53^

It might be objected that Sandel's worry isn't that genetic selection would make reproduction less unbidden, but that it would undermine our *appreciation* of the unbidden.

For this to make sense, reproduction and parenting must play a central, even constitutive role in our appreciation of a given world external to our will. But this is implausible. The genetic lottery didn't evolve in order to introduce chance into human life, or to induce humility in the face of the unbidden. It is there only because it is a more effective way to generate biological variation than asexual forms of reproduction.^54^

Might it nevertheless still be the case that, as Sandel claims, ‘parenthood, more than other human relationships, teaches … “an openness to the unbidden” ’?^55^ It is plausible enough that parenthood can teach us that, although it's more plausible that it does so by opening us to the unbidden nature of a child's developing *will*, not because it is unpredictable whether our child will have blue or brown eyes.

But let's concede that parenthood is one way to learn to appreciate the unbidden. But is it the only way, or even the central way? If it were, then this would mean that people who have no children have only a deficient sense of reality. Convents and monasteries remind us that religious tradition has little sympathy for this absurd suggestion. There are numerous ways to learn to appreciate a reality external to the self. Natural science, for example, is a paradigm of confrontation with the way things just happen to be, abstracted from anything human.

But even if we set this aside, there is simply no ground for thinking that genetic enhancement will undermine parents' appreciation of the unbidden, or that it expresses a vicious wilfulness.

Proponents of human enhancement often argue that we have reason to use genetic enhancement to bring into the world children with a range of talents and capacities most likely to lead to a good or flourishing life.^56^ To have such an aim is hardly to indulge in self-assertion. It is indeed a form of mastery, but it is mastery that is subservient to what is outside one's will: the welfare of a future person, and standards of the good life.^57^ If parents really wanted to brutely assert their arbitrary will, they would presumably choose a *random* set of traits — in other words, they would precisely *mimic* the natural genetic lottery!

It is doubtful that any parents will be wilful in this way, but no doubt there will be parents who will use reproductive technologies in misguided and shallow ways. This is hardly surprising. The availability of enhancement will not suddenly elevate people's moral character. But if the problem is with shallow values and attitudes, then it is not really with enhancement. Such shallow values and attitudes are expressed in numerous aspects of modern life. Instead of focusing on forbidding the use of technology, we should focus on changing these values and attitudes. Sandel complains that the use of genetic enhancement to promote human flourishing ‘deadens the impulse to social and political improvement’.^58^ This complaint oddly assumes that we cannot employ both means to these ends. But more importantly, the same complaint can be levelled at Sandel. For if his worry is really about our attitude to the unbidden, isn't this something that is also best addressed directly at the social level, rather than by preventing the use of technology?

In any case, genetic selection is actually likely to make prospective parents *more*, not less, acutely appreciative of the unbidden. In vitro fertilization is a highly demanding, unpleasant and uncertain process. And parents who use reproductive technologies to try to promote the wellbeing of their child will be engaged in a demanding project against a highly resistant reality. There is also the point that such parents would incur a great weight of responsibility. Ironically enough, Sandel himself sees this as an urgent worry.^59^ I don't deny that it is a worry. But it could not be the worry that such parents would lose touch with anything outside their will. On the contrary: the burden of moral responsibility, far from disconnecting us from a reality outside our wills, is a *paradigm instance* of such a reality, of the weight that, as Kundera puts it, makes our lives more real and significant.

It is a mistake, then, to identify support for human enhancement with a kind of satanic wilfulness, or with loss of an appreciation of an external reality. If we should be suspicious of anything, it's rather of the motivation that drives *opposition* to enhancement.

I said that the value of the unbidden is such that trying to actively promote it is, in a paradoxical way, partly self-defeating, because it is itself a form of mastery.^60^ But opposition to enhancement might be self-defeating in a further way, by itself expressing failure to accept the unbidden, and an unpleasant drive for mastery. For doesn't such opposition express precisely a desire to *master* technology and social change, to control the future — perhaps even to impose one's will, and fears, on others? If anything, it seems to me to express a desire to cling, not to unpredictability *per se*, but to a very predictable and *familiar* kind of unpredictability.^61^ Thus these worries, far from expressing openness to the unbidden, might in fact express deep fear of an unpredictable, risky and alien future — that is, fear of *losing* control.

A final word. We saw that the value of the unbidden is not just independent of theism, but not even fully compatible with it. And indeed a closer inspection of the Judeo-Christian tradition quickly reveals much that it is in tension with Sandel's argument against enhancement. After all, in the Old Testament, God gives his blessing, and active assistance, to Abraham and Sarah's pursuit of post-menopausal sex selection. And on most theist views, we are born with certain characteristics and talents precisely because *God* wills it so. God, then, could be said to select our genetic endowment. We are His artefacts, playing some role in His cosmic plan. If genetic selection involves a vicious attitude, what does that say about God?^62^

## 4. Conclusion

Sandel's argument against enhancement is unsuccessful. It is unsuccessful because, in several ways, he misunderstands the notions of mastery and the unbidden. Mastery isn't a kind of wilfulness, nor must it lead to a loss of a sense of anything external to the will; quite the contrary. And it is a mistake to identify the unbidden with the random and unpredictable. The necessary, and our moral obligations, are paradigms of the unbidden.

If the unbidden has value, it can only be fully realized in a naturalist world — it's in *tension* with a theist outlook.^63^ It is thus ironic that Sandel mistakenly identifies the unbidden with submission to a cosmic hierarchy that makes no sense on a naturalist worldview. Whether we ought to increase or reduce the unbidden in our lives has nothing to do with our attitude to some normative natural order. But if there *were* such a naturalorder which we ought to follow, then appeals to the unbidden, and denunciations of mastery, would be simply redundant.

These misunderstandings undermine Sandel's criticism of enhancement. I suspect that such anxiety about enhancement will one day seem as quaint as Goethe's fear that eyeglasses will corrupt relations between people, and that microscopes will disfigure our relation to nature.

Sandel remarks that ‘[t]he discovery that nature was not a meaningful order but a morally inert arena for the exercise of human will gave powerful impetus to the project of mastery.’^64^‘Discover’ is a factive verb; we cannot discover what isn't really there. It is thus odd that Sandel then adds that ‘[w]e may … have to choose between shaking off our unease with enhancement and finding a way beyond mechanism to the re-enchantment of nature.’^65^ This remark suggests that Sandel does not, in fact, believe that his argument can be detached from religion, or from a rejection of a naturalist worldview. Worse: to knowingly accept some comforting myth of the given, or pretend mysteries, would disfigure our relation to reality — it would be a spectacular failure to take a proper stance to the world, and to appreciate our true place in the universe.^66^

## Footnotes

1 Michael Sandel, *The Case Against Perfection* (Cambridge, MA: Harvard University Press, 2007), p. 9 (based on his ‘The case against perfection’, *The Atlantic Monthly* 293 (2004), reprinted in J. Savulescu & N. Bostrom (eds) *Human Enhancement* (Oxford: Oxford University Press, 2009)).

2 See Ronald Dworkin, ‘Playing God’, reprinted in his *Sovereign Virtue* (Cambridge, MA: Harvard University Press, 2002); Jürgen Habermas, *The Future of Human Nature* (Oxford: Polity Press, 2003).

3 See G. Kahane, ‘Should we want God to exist?’*Philosophy and Phenomenological Research* 82,3 (2011): 774–696; ‘Value and philosophical possibility’, forthcoming in *Philosophy and Phenomenological Research*.

4 It is not by accident that the debate about human enhancement cuts across familiar ethical and political divisions — anxiety about enhancement has been expressed by thinkers as diverse as Leon Kass, Jürgen Habermas and G. A. Cohen.

5 Wittgenstein famously said ‘I am not a religious man but I cannot help seeing every problem from a religious point of view’ (R. Rees (ed.) *Recollections of Wittgenstein* (Oxford: Oxford University Press, 1984, p. 79); Thomas Nagel, ‘Secular philosophy and the religious temperament’ in his *Secular Philosophy and the Religious Temperament* (Oxford: Oxford University Press, 2009). See also Robert Solomon, *Spirituality for the Skeptic* (Oxford: Oxford University Press, 2006).

6 Dworkin similarly distinguishes ‘derivative’ values such as wellbeing and autonomy, which are tied to the interests and rights of persons, and ‘detached’ values such as the sacredness of nature, that Dworkin also calls ‘religious’ (see e.g. R. Dworkin, *Life's Dominion* (New York: Vintage Books, 1994, p. 163). I don't think this is the best way to draw the distinction. Existential values *might* call for the kind of reverence which the religious associate with the sacred; but that is a substantive question that is best left open. It is also a substantive question whether existential values are purely ‘impersonal’ or rather play some significant role in our deepest interests.

7 Taken to extremes, this can lead to the implausible antirealist view that religious belief itself makes no metaphysical commitments, and is itself no more than a set of attitudes, values and practices.

8 Sandel 2004 op. cit., p. 57. See also Sandel 2007 op. cit., pp. 83–85, 100.

9 Sandel 2004 op. cit., p. 54.

10 See also Sandel 2007 op. cit., pp. 85–6, 93.

11 Frances Kamm, ‘What is and is not wrong with enhancement’, in J. Savulescu & N. Bostrom (eds) *Human Enhancement* (Oxford: Oxford University Press, 2009) is a formidable example of this approach.

12 Sandel's critique has clear affinities with the common complaint that it is wrong to ‘play God’— although Sandel studiously avoids using this phrase. Despite the explicit mention of God, that more familiar complaint is also often said to have force independently of religious belief. Thus Leon Kass writes that this complaint ‘is too facilely dismissed by scientists and nonbelievers. *The concern has meaning, God or no God*’ (Kass, ‘Triumph or tragedy? The moral meaning of genetic technology’, *American Journal of Jurisprudence* 45 (2000): 1–16, at p. 9; my italics). Much of what I will say against Sandel also has force against this view. I am grateful here to the editors.

13 Political philosophers call this ‘brute luck’. Sandel speaks both of the unbidden and of ‘the given’, but he uses these interchangeably (see Sandel 2007 op. cit., p. 93). I will reluctantly follow Sandel in speaking about ‘the unbidden’, even though its heavy biblical resonance also makes it rather opaque. But the alternatives aren't better: ‘the given’ has irrelevant philosophical connotations, while ‘chance’ and ‘luck’ are unhelpfully associated with unpredictability.

14 Though admittedly the unpredictable is *harder* to master, and unpredictability indicates a limit to our *cognitive* mastery. Note that most things are random and unpredictable only relative to our limited epistemic situation. But according to contemporary physics, indeterminism might inhere in the very nature of things.

15 Conversely, what is under someone's control might be utterly unpredictable — even more so on some libertarian views of free will.

16 Thus Boethius wrote that ‘[i]f chance is defined as an event produced by random motion and without any sequence of causes, then I say that there is no such thing as chance … For what room can there be for random events since God keeps all things in order?’*The Consolations of Philosophy*, book V. See also Augustine, *City of God*, v. 9. If anything, an attitude of somber acceptance of the unbidden would make better sense in the context of ancient Greek thought, for which the idea of an ineluctable fate (*heimarmenê*) was central.

17 The problem of evil is famously generated by this implication of theism. But note that even if God were not ultimately responsible for the choices of free agents, it would still remain the case that in a Godly world *some* agent is ultimately responsible for *everything* that happens.

18 If the naturalist universe is deterministic, and determinism is incompatible with free will, then perhaps in a naturalist universe *everything* is absolutely unbidden!

19 For a fascinating discussion of the way lots were used by 18th century Moravian missionaries in the North American frontier, see Christopher Gavaler, ‘The empty lot: Spiritual contact in Lenape and Moravian religious beliefs’, *American Indian Quarterly* 18 (1994): 215–228. The nastier medieval practice of trial by ordeal similarly appealed to the idea of *judicium dei*.

20 They go against Christian orthodoxy: Augustine objected to the use of lotteries precisely because they ‘tempt God’.

21 This is why religious believers are sometimes warned that it is wrong to wish people ‘good luck’. See James Wood, ‘Everything, something, nothing: The modern novel and the new atheism’, *ABC Religion and Ethics*, First Posted 4 July 2011 at http://www.abc.net.au/religion/articles/2011/07/04/3259863.htm

22 There is a tradition within monotheism — think of the Book of Job, or of Kierkegaard — which sees God's will as inscrutable, even arbitrary. This tradition also encourages submission to God's will, *whether or not* we can see it as unambiguously good. But even on this tradition nothing is absolutely unbidden in the above sense. I am grateful here to an anonymous reviewer.

23 Augustine, *City of God*, iv. 18–18, v. 8.

24 Similar considerations are behind influential accounts of distributive justice. How could it be fair that one person has a far worse life, and far fewer opportunities, than another, just because she was born to disadvantaged parents — let alone, just because she was born with a less promising genetic endowment? The whole luck egalitarian tradition is based on the idea that it's unjust to leave the distribution of goods to pure chance, because differences in possession of goods should reflect only the consequences of choice. (This is compatible with the point that, when several parties has equal claim on a good that cannot be equally divided, we can use a lottery to justly decide who should get it.)

25 There is in addition the important point that freedom of the will and its exercise — that is to say *mastery*— are also greatly valuable in themselves, a claim central to many theist views. And it is a *further* good when what is good occurs not randomly but *because* it is good, and recognized as such. The theist universe is in this respect more attractive to many precisely because the good in it exists *because* it is good, and not through cosmic accident. Finally, it is a central insight of recent epistemology that luck undermines *knowledge*— that is, *cognitive mastery*. See Duncan Pritchard, *Epistemic Luck* (Oxford: Clarendon Press, 2005).

26 Sandel 2007 op. cit., p. 100.

27 We can of course also master ourselves, but precisely as something resistant to the will. See Harry Frankfurt, ‘The faintest passion’, *Necessity, Volition and Love* (Cambridge: Cambridge University Press, 1999).

28 Descartes denied this, but if true, then the claim that nothing is absolutely unbidden if God exists need to be qualified, since many *necessary* truths would be independent of God's will, and thus unbidden even if theism is true. This qualification does not affect my argument.

29 This point holds even on metaethical views that tie morality to human dispositions and concerns. Our deepest concerns are not something we can simply decide at will.

30 Johan Gottlieb Fichte, *Foundations of Natural Right* (Cambridge: Cambridge University Press, 1796/2000).

31 Sigmund Freud, ‘Formulations on the two principles of mental functioning’ in J. Strachey (ed.) *The Standard Edition of the Complete Psychological Works of Sigmund Freud*, vol. 12 (London: The Hogarth Press, 1958).

32 Weil writes that ‘[a] test of what is real is that it is hard and rough. Joys are found in it, not pleasure. What is pleasant belongs to dreams’ (*Gravity and Grace* (London: Routledge, 1947/2002), p. 53). And Frankfurt writes that ‘[t]he concept of reality is fundamentally the concept of something which is independent of our wishes and by which we are therefore constrained’ (op. cit., p. 100). See also John Henry Newman, *Grammar of Assent*, 1.4.1; Bernard Williams, *Truth and Truthfulness* (Princeton, NJ: Princeton University Press, 2004), pp. 125–126 and Robert Nozick, ‘Happiness’, in his *The Examined Life* (New York: Simon and Schuster, 1989).

33 Milan Kundera, *The Unbearable Lightness of Being* (New York: Harper & Row, 1984), p. 5.

34 Needless to say, the spoiled child has *mastered nothing*; it is only a rudimentary agent. By contrast, the wilful person *does* exhibit (to borrow Sandel's phrase) a kind of hyperagency, a desire to impose his will on the world — the spoiled child fails to even recognize the distinction between his will and reality. But both narcissistically see everything around them only through the prism of their own desires; and perhaps the wilful person was once a spoiled child …

35 Jean Amery, *At the Mind's Limits* (Bloomington, IN, Indiana University Press, 1980), p. 19.

36 A character in a Phillip Roth novel similarly describes refusal to accept that a tragedy was simply ‘pointless, contingent, preposterous’ as a form of *hubris*, ‘not the hubris of will or desire but the hubris of fantastical, childish religious interpretation’: *Nemesis* (New York: Random House, 2010), p. 246.

37 See K. Laurin, A. C. Kay & D. M. Moscovitch, ‘On the belief in God: Towards an understanding of the emotional substrates of compensatory control’, *Journal of Experimental Social Psychology* 44 (2008): 1559–1562; A. C. Kay, D. M. Moscovitch & K. Laurin, ‘Randomness, attributions of arousal, and belief in God’, *Psychological Science* 21 (2010): 216–218.

38 Wallace Stevens, ‘Of Mere Being’, *Collected Poems* (New York: Knopf, 1954).

39 Richard Dawkins, *River Out of Eden* (New York: Basic Books, 1996), p. 133.

40 These speculative remarks raise philosophical (and theological) issues that I cannot fully address here — but they aren't required for my criticism of Sandel in the next section. It's worth distinguishing, however, the claim that if in fact God *doesn't* exist, there is great value in facing up to the absolutely unbidden nature of the universe we inhabit (and that belief in God might be a way of avoiding this disturbing truth), and the far stronger claim that a Godless universe is *better* because it allows us to face something absolutely unbidden.

41 *Pace* Solomon op. cit., and Michael Hauskeller, ‘Human enhancement and the giftedness of life’, *Philosophical Papers* 40,1: 55–79. In other words, just as it cannot make sense for an atheist to pray to a God above, it cannot make sense to treat life as *literally* a gift. Such practices and attitudes *aren't* theism-neutral. This isn't to deny that when a great good unexpectedly lands in our hands, this can occasion a distinctive kind of gladness; we can call such gladness ‘gratitude’ if we wish, but this is more likely to mislead than to illuminate.

42 My claim is that this value can be realized only in a qualified way if God exists, not that it can't be realized *at all*. Consider the parallel example of privacy. If an omniscient God exists, then nothing we think, feel or do is ever *absolutely* private, known *only* to us; we can only enjoy privacy relative to other mortals (see Kahane 2011 op. cit.). There are similarly many ways in which we can come to recognize limits to our will — by recognizing, for example, the will of others, and, in a theist universe, the will of God. But another's will — even with the will of God — confronts us only with what is unbidden *relative* to us. It isn't the same as confrontation with what I called the *absolutely* unbidden, with what simply occurs, without sense or purpose. Such a confrontation is impossible if God exists. Even if we thought that God's will is inherently inscrutable, perhaps even arbitrary, we would still experience the universe as a place in which everything that happens can be traced to someone's agency. I'm grateful here to an anonymous reviewer.

43 Though notice that this worry is generated not by the mastery involved in *developing* such technology, but by the effortless use of some of the *products* of such mastery.

44 Chance that is under our mastery in this way is, in Dworkin's terms, ‘option luck’, not brute luck.

45 This is not something we ought to regret. As I've argued elsewhere, one of the less attractive aspects of theism is that it presents us as subservient to a superior, and His master plan, rather than as fully autonomous beings with equal status. See Kahane 2011 op. cit.

46 And like theism, it can also be viewed as driven by refusal to accept the utterly unbidden contingency and arbitrariness of the natural world.

47 Russell Powell and Allen Buchanan, ‘Breaking evolution's chains’, in J. Savulescu, R. ter Meulen & G. Kahane (eds) *Enhancing Human Capacities* (Oxford: Wiley-Blackwell, 2011).

48 See e.g. Philip Kitcher, ‘Essence and perfection’, *Ethics* 110 (1999): 59–83; Dale Dorsey, ‘Three arguments for perfectionism’, *Noûs* 44 (2010): 59–79; Tim Lewens, ‘Foot note’, *Analysis* 70 (2010): 468–473. See also Tim Lewens, ‘Enhancement and human nature: The case of Sandel’, *Journal of Medical Ethics* 35 (2009): 354–356, for criticism of Sandel's understanding of nature.

49 Indeed, these two sources of value are ultimately incompatible. For suppose that the given world around us radically departs from the norm of the natural order. To be open to the unbidden, we would have to accept what we have been ‘given’; to follow the natural order, we would need to radically change things.

50 In conversation, Sandel appeared to concede that even traditional arranged marriages are superior in this respect to current arrangements, since they impose the unbidden on the couple (even though they aren't *absolutely* unbidden in my sense). And notice that the modern practice of freely choosing one's romantic partner couldn't be plausibly defended by appeal to what is ‘natural’.

51 Notice that since I am here drawing the implications of Sandel's own claims, I'm now ignoring the distinction between the unbidden and unpredictable; the policies I describe will in any case increase both. As noted earlier, it's partly self-defeating to try to implement policies to increase the unbidden — and this problem also afflicts attempts to constrain the development and use of reproductive technology. I return to this below.

52 Given that Sandel's objection to enhancement explicitly brackets questions about the welfare of the resulting child, I'll ignore the important distinction between genetic selection (from a pool of embryos) and genetic enhancement (of some particular child).

53 Indeed the contrast between mastery and the unbidden can be misleading, because it obscures the truism that the exercise of mastery often has numerous unintended (viz. unbidden) consequences — consequences that are often *far less* predictable than leaving things to their natural course (just think of global warming).

54 Reproduction can seem a sacred mystery only to those who wilfully ignore modern biology. We now know, for example, that many seemingly innocent aspects of human reproduction in fact reflect a fiercely competitive evolutionary history. One recent study even suggests that the shape of the penis of the human male was selected to displace the semen of competing males. See Gordon Gallup, R. Burch, M. Zappieri, R. Parvez, M. Stockwell, & J. Davis, ‘The human penis as a semen displacement device’, *Evolution and Human Behaviour* 24 (2003): 277–289.

55 Sandel 2007 op. cit., p. 45.

56 See e.g. Jonathan Glover, *Choosing Children* (Oxford: Oxford University Press, 2004). My argument requires only that enhancement will be driven by such concern for the future child's wellbeing. It is compatible with, but doesn't require, the far stronger view that genetic selection should be used to create the *best* possible child (cf. Julian Savulescu & Guy Kahane, ‘The moral obligation to create children with the best chance of the best life’, *Bioethics* 23 (2009): 274–290.)

57 For a similar point, see Kamm op. cit.

58 Sandel 2007 op. cit., p. 97.

59 Sandel 2007 op. cit., p. 89. I ignore here Sandel's further worry that enhancement will undermine solidarity, since it is based on psychological speculations that are supported by no evidence whatsoever.

60 Sandel's worries about enhancement are strikingly similar to Heidegger's critique of technology (Hans Jonas and Hanna Arendt might be mediating influences). But Heidegger already recognized that the desire to control technology is self-defeating; ironically, Heidegger describes *this* desire as a ‘will to mastery’. See ‘The question concerning technology’ in *The Question Concerning Technology and Other Essays* (New York: Harper Torchbooks, 1977), p. 5.

61 Indeed, as we have seen, if what was valued was really unpredictability, there are numerous ways in which reproduction could be made less predictable (and more unbidden). Indeed, it would be easy to make human life as a whole less predictable: J. L. Borges's story ‘The Lottery in Babylon’ describes a society in which every aspect of life is decided by a secret lottery (*Labyrinths: Selected Stories* (Harmondsworth: Penguin Modern Classics, 2000)); in Luke Reinhardt's cult novel *The Dice Man* (New York: HarperCollins, 1999), a doctor surrenders all life decisions to the roll of a dice.

62 Habermas argues that children who were genetically enhanced would not possess genuine autonomy and self-consciousness; human autonomy can exist only so long as natural reproduction is unpredictable or, if you wish, absolutely unbidden (op. cit., p. 13). Like Sandel, Habermas also sees his critique of enhancement as drawing on religious sentiments. But like Sandel's it is in fact incompatible with theism. For it seems to follow from this view that if God exists, then our genetic endowment has been selected by God, and no one is in fact autonomous. I don't see how Habermas can block this implication.

63 As an anonymous reviewer reminded me, it may not be in tension with non-theistic religions such as Buddhism or Daoism. Karma, for example, often represents a purely impersonal moral order and can thus count as absolutely unbidden, in my sense. It might also not be in tension with forms of theism that offer a radically impersonal understanding of God.

64 Sandel, ‘What's wrong with enhancement’, paper presented to the President's Council in Bioethics (2002). See http://bioethics.georgetown.edu/pcbe/background/sandelpaper.html (accessed 26 October 2010).

65 Sandel 20002 op. cit.

66 I am grateful to the organizers of the *Reason, Theology and the Genome* conference at Christ Church College, Oxford, for which this paper was originally prepared. I greatly benefitted from suggestions by the audience at that conference, and an audience at the 3TU research day at Utrecht. I am especially grateful to suggestions by Alexandra Couto, Stefan Koller, Martin Peterson, an anonymous reviewer, and the editors, and for very helpful comments by Michael Haukskeller. Work on this paper was generously supported by a University Award from the Wellcome Trust (*WT087208MF*).

